# Miglustat Reverts the Impairment of Synaptic Plasticity in a Mouse Model of NPC Disease

**DOI:** 10.1155/2016/3830424

**Published:** 2016-01-14

**Authors:** G. D'Arcangelo, D. Grossi, M. Racaniello, A. Cardinale, A. Zaratti, S. Rufini, A. Cutarelli, V. Tancredi, D. Merlo, C. Frank

**Affiliations:** ^1^Department of Medical System, University of Rome Tor Vergata, 00133 Rome, Italy; ^2^Department of Cell Biology and Neuroscience, Istituto Superiore di Sanità, 00161 Rome, Italy; ^3^IRCCS San Raffaele Pisana, 00163 Rome, Italy; ^4^Department of Biology, University of Rome Tor Vergata, 00133 Rome, Italy; ^5^National Centre for Rare Diseases, Istituto Superiore di Sanità, 00161 Rome, Italy

## Abstract

Niemann-Pick type C disease is an autosomal recessive storage disorder, characterized by abnormal sequestration of unesterified cholesterol within the late endolysosomal compartment of cells and accumulation of gangliosides and other sphingolipids. Progressive neurological deterioration and insurgence of symptoms like ataxia, seizure, and cognitive decline until severe dementia are pathognomonic features of the disease. Here, we studied synaptic plasticity phenomena and evaluated ERKs activation in the hippocampus of BALB/c NPC1−/− mice, a well described animal model of the disease. Our results demonstrated an impairment of both induction and maintenance of long term synaptic potentiation in NPC1−/− mouse slices, associated with the lack of ERKs phosphorylation. We then investigated the effects of Miglustat, a recent approved drug for the treatment of NPCD. We found that* in vivo* Miglustat administration in NPC1−/− mice was able to rescue synaptic plasticity deficits, to restore ERKs activation and to counteract hyperexcitability. Overall, these data indicate that Miglustat may be effective for treating the neurological deficits associated with NPCD, such as seizures and dementia.

## 1. Introduction

Niemann-Pick type C disease (NPCD) is a panethnic, fatal, autosomal recessive, neurovisceral lipid storage disorder with infantile and juvenile onset in 95% of cases and adult onset in 5% of cases [1]. The NPC genes,* NPC1* mutated in about 95% of the disease and* NPC2* mutated in the remaining 5%, encode proteins that are involved in intracellular lipid transport. Mutations in* NPC1* and* NPC2* give rise to severe abnormalities in the functioning of this transport system with excess storage of lipids as cholesterol, glycosphingolipids, and sphingosine in the late endosomal and lysosomal intracellular compartments, associated with peripheral and central organ dysfunction [2].

The disease is characterized by progressive neurological deterioration and the insurgence of symptoms like ataxia, seizure, and cognitive decline until severe dementia leading to premature death [2, 3]. Although the genetic defects causing NPCD are well known, very little information is available on the causes of neurological deficits and neuropathology. Current therapeutic approaches for NPCD are limited. N-butyldeoxynojirimycin (Miglustat; Zavesca, Actelion Pharmaceuticals), a drug initially used for the treatment of several dyslipidosis including GM1 gangliosidosis, Gaucher type I, and Tay-Sachs disease, has been approved in 2009 for the treatment of progressive neurological manifestations in adult and pediatric patients affected by NPCD [4–7]. The active principle of the drug is an iminosugar able to specifically inhibit the enzyme glucosylceramide synthase (GCS) that converts the ceramide into the glycosphingolipid glucosylceramide (i.e., the first product in the synthesis of complex glycosphingolipid, including gangliosides). Although undeniable advantages concerning some neurological symptoms have been noticed in clinic, the reason why cells presenting a bias in the transport system of both cholesterol and all classes of sphingolipids should benefit from the block of glycolipids synthesis is not clear [8].

Changes in the plasma membrane cholesterol content and in the glycosphingolipids/cholesterol ratio are particularly important in affecting lipid rafts, platforms where many transductive signaling processes are generated, fundamental for a variety of cellular functions and important regulators of glutamate receptor activity [9, 10]. We demonstrated in a previous paper that cholesterol depleted hippocampal neurons exhibit an impaired NMDA receptors-mediated synaptic plasticity [11]. Therefore, alteration of a correct dynamic of cholesterol-sphingolipids content in the neuronal plasma membrane might have a key role in neuronal dysfunction associated with NPC disease, such as memory impairment and dementia.

Aim of this study is to evaluate whether synaptic plasticity phenomena, involved in learning and memory processes, are affected in NPC1−/− mice, a well-established mouse model for the NPC disease, and if* in vivo* Miglustat treatment is able to counteract synaptic deficits. To this aim, we employed a multidisciplinary approach both in Wild Type (WT) and in NPC1−/− mice, consisting in (i) electrophysiological recording in acute hippocampal slices and (ii) molecular analysis of intracellular pathway.

## 2. Methods

### 2.1. Animals

We first established a colony of NPC1−/− mice that represent a well-known experimental model of NPCD since they display most of the clinical features of the disease including cognitive deficits. Breeding pairs of BALB/cNctr-Npc1m1N/J (Stock number: 003092) mice were purchased from Jackson Laboratories (Bar Harbor, MA, USA). This strain contains a spontaneous mutation in the NPC1 locus [12]. Animals were bred and maintained according to Italian Animal Care Committee rules. Heterozygous (male × female) mice were bred and the genotypes of offspring animals were determined as indicated by Jackson Laboratories in genotyping protocols database by polymerase chain reaction (PCR) [12]. Briefly, PCR was performed using the following primers: WT sense: CTG TAG CTC ATC TGC CAT CG, WT antisense: TCT CAC AGC CAC AAG CTT CC; mutant sense: GGT GCT GGA CAG CCA AGT A, mutant antisense: TGA GCC CAA GCA TAA CTT CC. The expected products were as follows: mutant = 475 bp, WT = 173 bp, heterozygote = 173 bp, and 475 bp. Age-matched WT served as controls.

### 2.2. Miglustat Administration

The experiments have been performed to study the effects of* in vivo* Miglustat (N-butyldeoxynojirimycin, Sigma) treatment on NPC1−/− mice synaptic activity. In addition to WT mice (*n* = 12) and NPC1−/− untreated mice (*n* = 11), we distributed the treated animals in four groups: WT mice treated by oral administration (gavage) with either saline solution (*n* = 5) or Miglustat (0.2 mg/kg) (*n* = 5) and NPC1−/− mice treated by oral administration (gavage) with either saline solution (*n* = 5) or Miglustat (0.2 mg/kg) (*n* = 11).

NPC1−/− mice showed well-defined symptoms consisting in ataxia and intentional tremor since 50–52 days of age. Before the insurgence of the characteristic symptoms of the pathology (the 38th day of life), NPC1−/− and WT mice have been treated for 20 days. During this temporal window, no gastroenteric side effects linked to immino sugar administration were evident and they were able to feed and did not show insurgence of neurological symptoms presenting a normal function motor system (see Video in Supplementary Material available online at http://dx.doi.org/10.1155/2016/3830424). Because we aimed at evaluating the effects of Miglustat on synaptic plasticity deficits rather than its general effects on clinical symptoms and lifespan, animals were sacrificed at the end of treatment, at 58–60 days of life.

### 2.3. Extracellular Recordings in Mouse Hippocampus

BALB/cNctr-npc1N mice, genotypically characterized littermates (5–7 weeks old), were used according to the procedures established by the European Union Councils of Animal Care European Communities Council Directive of 24 November 1986 (86/609/EEC). All efforts were made to minimize the number of animals used and their suffering. Under anesthesia with enflurane, they were decapitated and brains were quickly removed and placed in cold, oxygenated artificial cerebral spinal fluid (ACSF) containing the following (in mM): NaCl 124, KCl 2, KH_2_PO_4_ 1.25, MgSO_4_ 2, CaCl_2_ 2, NaHCO_3_ 26, and glucose 10 [13]. The hippocampus was rapidly dissected and slices (450 *μ*m thick) were cut transversely with a McIlwain tissue chopper (Mickle Laboratory Engineering Co., Gomshall, UK) and transferred into a tissue chamber, where they were laid in an interface between oxygenated ACSF and humidified gas (95% O_2_/5% CO_2_) at 32–34°C (pH = 7.4), constantly superfused at flow rate of 1.2 mL/min. Extracellular recordings of the population spikes (PSs) were made in the stratum pyramidale of the CA1 subfield with glass microelectrodes filled with 2 M NaCl (resistance 5–10 MΩ). Orthodromic stimuli (10–500 mA, 20–90 ms, 0.1 Hz) were delivered through a platinum electrode placed in the stratum radiatum in the Schaffer collateral/commissural CA1 pathways. The test stimulus intensity of 50 ms square pulses was adjusted to elicit a PS of 2-3 mV at 0.03 Hz. PS amplitude was calculated every minute as the average of six recordings performed every 10 s. To exploit basal synaptic transmission, the PS was recorded for 1 hour. After recording stable signals (20–30 min), a tetanic stimulation (100 Hz, 1 s) was delivered to induce long term potentiation (LTP) at the same stimulus intensity used for the baseline responses. Posttetanic potentiation (PTP) and LTP were measured by calculating the PS amplitude prior to and after the tetanus.

Field potentials were fed to an Axoclamp 2A amplifier, acquired through a digital/analogic system (Digidata 1440A, Axon Instruments) and analyzed with the software pCLAMP10 (Axon, Foster City, CA, USA).

Changes in the amplitude of PS after tetanization were expressed as percentages of the basal PS amplitude (PS/PS_basal_ *∗*100, where PS_basal_ is the mean PS amplitude before tetanization). The overall effects on PTP and LTP were measured by calculating the average, for each slice, of the PS amplitudes recorded during the 5 min period before the tetanus (BST), during the first minute after the tetanus (PTP), and during the 60 min period after the tetanus (LTP).

### 2.4. Immunoblotting Assays

Hippocampal slices used for electrophysiological experiments were rapidly dissected to isolate CA1 regions and then homogenized in ice-cold RIPA buffer containing 1x complete protease and phosphatase inhibitors (Sigma). Protein concentration was assessed by the Micro BCA Protein Assay Kit (Pierce). Proteins (30 *μ*g) were subjected to SDS-polyacrylamide gel electrophoresis (SDS-PAGE) on polyacrylamide gels and electrophoretically transferred to nitrocellulose membranes as previously described [14]. Loading of protein samples was verified by Coomassie and Ponceau staining. Primary antibodies directed against phospho-p44/p42 MAPK (Thr202/Tyr204), total p44/p42 MAPK were obtained from Cell Signaling Technology. Membranes were first processed to visualize the phosphorylated forms of proteins, dehybridized (Restore Western Blot Stripping Buffer, Pierce, Rockford, IL, USA), and then reprobed with antibodies directed against total proteins for normalization. Quantitation was carried out by densitometric film analysis.

### 2.5. Statistics

For electrophysiological experiments, data are expressed as mean measurements ± SEM and *n* represents the number of slices studied. Data were compared with Student's *t*-test or the ANOVA test and were considered significantly different if *p* < 0.05. Excel 5.0 software was used for generation of graphs. For ERKs, activation studies data, expressed as mean ± SEM, were statistically analyzed using Student's *t*-test.

## 3. Results

### 3.1. Effect of Miglustat on Basal Synaptic Transmission

In a previous paper, we demonstrated that basal synaptic transmission (BST) recorded in the CA1 region of hippocampal slices from NPC1−/− mice was enhanced with respect to slices from WT mice [15]. In the present study, we analyzed whether* in vivo* Miglustat administration could be able to reduce synaptic transmission in NPC1−/− mice.

**Figure 1:**
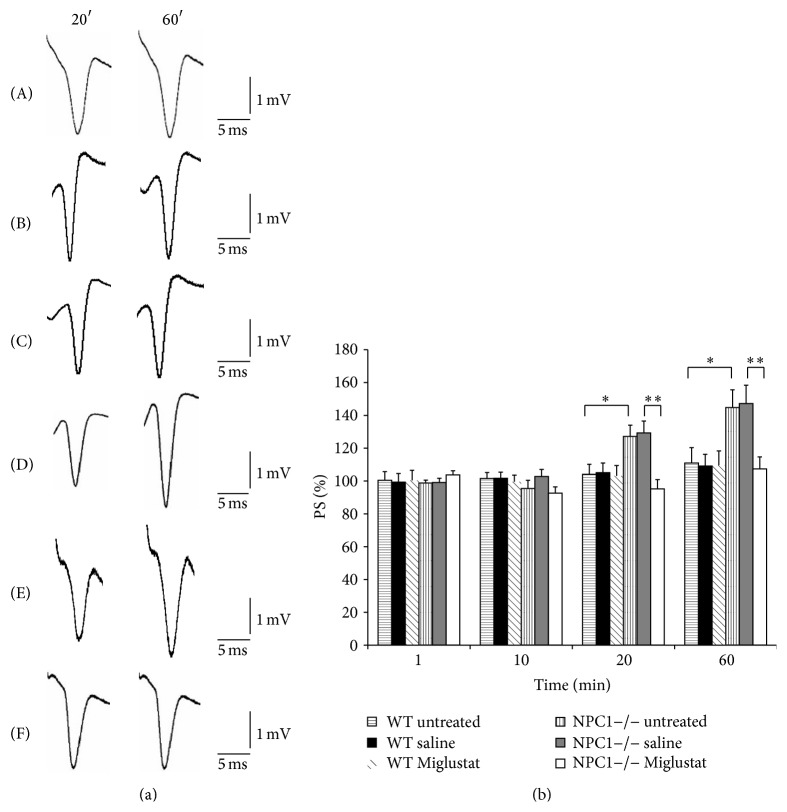
Basal synaptic transmission in CA1 hippocampal subfield in slices obtained from WT and NPC1−/− mice. (a) Recordings were acquired at times *t* = 20 and *t* = 60: curves (A), (B), and (C) refer to WT untreated, WT saline treated, and WT Miglustat treated mice slices, whereas curves (D), (E), and (F) represent the PS of NPC1−/− untreated, NPC1−/− saline, and NPC1−/− Miglustat treated mice. (b) % PS amplitude as a function of time is shown in WT untreated (horizontal pattern bar, *n* = 8), in WT saline treated (black bar *n* = 5), in WT Miglustat treated (diagonal pattern bar, *n* = 5), in NPC1−/− untreated (vertical pattern bar, *n* = 8), in NPC1−/− saline treated (grey bar, *n* = 5), and in NPC1−/− Miglustat treated (white bar, *n* = 11) mouse slices at minutes 1, 10, 20, and 60. PS amplitude corresponds to an average of 6 recordings/min. Bars in the plot are means ± SEM of values obtained from different slices. Note significant statistical differences in PS amplitude during 1-hour recording: at 20 min recording 127.1 ± 6 versus 104.1 ± 6.1, respectively, in NPC1−/− and WT slices of untreated mice (^*∗*^ *p* < 0.05) and 95.2 ± 5.6 versus 129.4 ± 7.2, respectively, in NPC1−/− slices of Miglustat and NPC1−/− slices of saline treated mice (^*∗∗*^ *p* < 0.01); at 60 min recording 144.7 ± 10 versus 111 ± 9 in NPC1−/− and WT slices of untreated mice (^*∗∗*^ *p* < 0.01) and 107.3 ± 7.3 versus 147.2 ± 11 in NPC1−/− slices, respectively, of Miglustat and saline treated mice (^*∗∗*^ *p* < 0.01).

In NPC1−/− and WT slices from untreated animals, PS % values at 20 min of the recording time period of BST were 127.1 ± 6 and 104.1 ± 6.1 whereas at 60 min they were 144.7 ± 10.8 and 111 ± 9.3, respectively (*n* = 8 from 8 different animals for each group, *p* < 0.05, Figure 1). In slices from WT animals treated with saline solution (*n* = 5 from 5 different animals) and with Miglustat (*n* = 5 from 5 different mice), the BST values did not significantly change compared to the untreated mice (105.1 ± 5.9 and 103.2 ± 6.3, resp., versus 104 ± 6.1 at 20 min; 109.1 ± 7.1 and 109.7 ± 8.7 versus 111 ± 9.3 at 60 min, Figure 1), thus excluding any event due to gavage technique or Miglustat unspecific effects. Indeed, in slices from NPC1−/− mice treated with saline solution (*n* = 5 from 5 different animals), the PS values did not vary with respect to the untreated (129.4 ± 7.2 versus 127.1 ± 6 at 20 min; 147.2 ± 11.2 versus 144 ± 10.8 at 60 min). After 20 days of oral Miglustat administration, PS recorded in the CA1 region of hippocampal slices from NPC1−/− mice (*n* = 11 from 11 different animals) remained stable for all the recording time compared to NPC1−/− saline treated mice: value at 20 min 95.2 ± 5.6 versus 129.4 ± 7.2 (*p* < 0.01) and value at 60 min 107.4 ± 7.3 versus 147.2 ± 11.2 (*p* < 0.01), indicating a counteracting effect of the drug on the hyperexcitability observed in the NPC1−/− mice (Figure 1).

### 3.2. Impairment of Synaptic Plasticity in CA1 Hippocampal Region of NPC1−/− Mice Was Rescued by Miglustat Treatment

**Figure 2:**
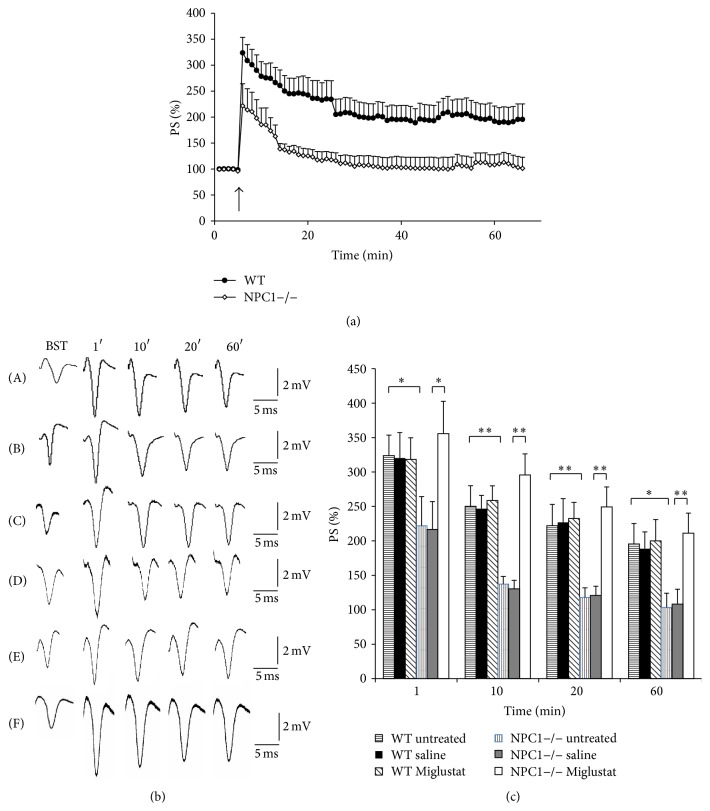
Synaptic plasticity in CA1 hippocampal subfield of WT and NPC1−/− mice. (a) % PS amplitude as a function of time is shown after tetanic stimulation applied at time *t* = 5 (arrow). The PS amplitude, measured every minute, corresponds to an average of 6 recordings/min. Points in the plot are means ± SEM of values obtained from different slices. Note the impairments of both the induction and the expression of LTP in NPC1−/− untreated mice (white rombs, *n* = 11) in comparison to the WT (black circles, *n* = 12) untreated mice. (b) Recordings were obtained from different slices of WT (A) and NPC1−/− untreated (B), WT saline (C) and NPC1−/− saline treated (D), and WT Miglustat (E) and NPC1−/− Miglustat (F) treated mice. The first curve of each group refers to the BST and it was recorded before the application of the tetanic stimulation, while the other curves refer to population spikes at times 1, 10, 20, and 60 min after the HFS. (c) % PS amplitude after HFS as a function of time is shown in WT untreated (horizontal pattern bar, *n* = 12), in WT saline treated (black bar *n* = 5), in WT Miglustat treated (diagonal pattern bar, *n* = 5), in NPC1−/− untreated (vertical pattern bar, *n* = 11), in NPC1−/− saline treated (grey bar, *n* = 5), and in NPC1−/− Miglustat treated (white bar, *n* = 11) mice slices at minutes 1, 10, 20, and 60. Bars in the plot are means ± SEM of values obtained from different slices. Note significant statistical differences in PS amplitude during 1-hour recording in NPC1−/− untreated versus WT untreated mice slices: PTP and LTP values at 10, 20, and 60 min after tetanic stimulation were, respectively, 221.8 ± 42.5, 137.3 ± 11, 117.8 ± 14, and 103.3 ± 20.9 versus 323.8 ± 29.8, 250.2 ± 29.9, 222 ± 31.9, and 195.6 ± 29.8 (^*∗*^ *p* < 0.05 at 1 and 60 min; ^*∗∗*^ *p* < 0.01 at 10 and 20 min). A statistically significant difference is also present in NPC1−/− Miglustat treated versus NPC1−/− saline treated mice slices: the values recorded were 355.7 ± 46.8, 295.8 ± 30.5, 249.4 ± 29, and 211.1 ± 29.1 versus 216.7 ± 40.4, 130.3 ± 12.4, 120.8 ± 13.5, and 108.3 ± 21.4 (^*∗*^ *p* < 0.05 at 1 min; ^*∗∗*^ *p* < 0.01 for all the following minutes).

First we evaluated synaptic plasticity in CA1 hippocampal region of NPC1−/− and WT mice because memory loss is one of the pathognomonic symptoms of NPC disease. For this purpose, we induced long term potentiation (LTP) at Schaffer collateral/commissural fiber-CA1 synapses in hippocampal slices using one train of high frequency stimulation (HFS) (1 s 100 Hz). This stimulation induced a sustained enhancement of PS in WT mice (Figure 2(a)): the values of PTP were 323.8 ± 29.8 while the values of the LTP recorded at 10, 20, and 60 min after the tetanus were 250.2 ± 29.9, 222 ± 31.9, and 195.6 ± 29.8, respectively (*n* = 12 from 12 different animals) (Figure 2(c)). The same stimulation in the NPC1−/− mouse slices induced a marked inhibition of the expression of both PTP and LTP and a complete blockade of the LTP maintenance phase. PTP and LTP values at 10, 20, and 60 min after tetanic stimulation were 221.8 ± 42.5, 137.3 ± 11, 117.8 ± 14, and 103.3 ± 20.9, respectively (*n* = 11, *p* < 0.05 and *p* < 0.01 versus untreated WT; see the legend for details). The decrease of PTP was approx. 32% whereas the LTP decrement was about 45%, 50%, and 47% at 10, 20, and 60 min of recording, respectively.

In slices from WT animals treated with saline solution (*n* = 5 from 5 different animals) and with Miglustat (*n* = 5 from 5 different mice), the PTP and LTP values at 10, 20, and 60 min after tetanic stimulation did not significantly change with respect to the untreated mice. The values were 320.3 ± 36.9, 246.7 ± 19.3, 226.9 ± 34.5, and 188.6 ± 24.4, respectively, for the WT saline treated mice and 318.3 ± 31.2, 258.6 ± 21.3, 232.6 ± 23.1, and 200.1 ± 30.8 for the WT Miglustat treated mice. In slices from NPC1−/− mice treated with saline solution (*n* = 5 from 5 different animals), tetanic stimulation induced an inhibition of both PTP and LTP as reported for the NPC1−/− untreated mice. The PS % values were 216.7 ± 40.4 for PTP and 130.3 ± 12.4, 120.8 ± 13.5, and 108.3 ± 21.4 at 10, 20, and 60 min after tetanic stimulation.

Miglustat treatment in the NPC1−/− mice was able to revert the impairment of both PTP and LTP observed in slices from NPC1−/− saline treated mice. The values of PTP and LTP at 10, 20, and 60 min after tetanic stimulation were 355.7 ± 46.8, 295.8 ± 30.5, 249.4 ± 29, and 211.1 ± 29.1, respectively, thus reaching values similar to those of saline treated WT mice (*n* = 11, *p* < 0.05 and *p* < 0.01 versus saline treated NPC1−/−; see the legend for details).

### 3.3. Effect of Miglustat on ERK Phosphorylation in Hippocampal Slices of NPC1−/− Mice

**Figure 3:**
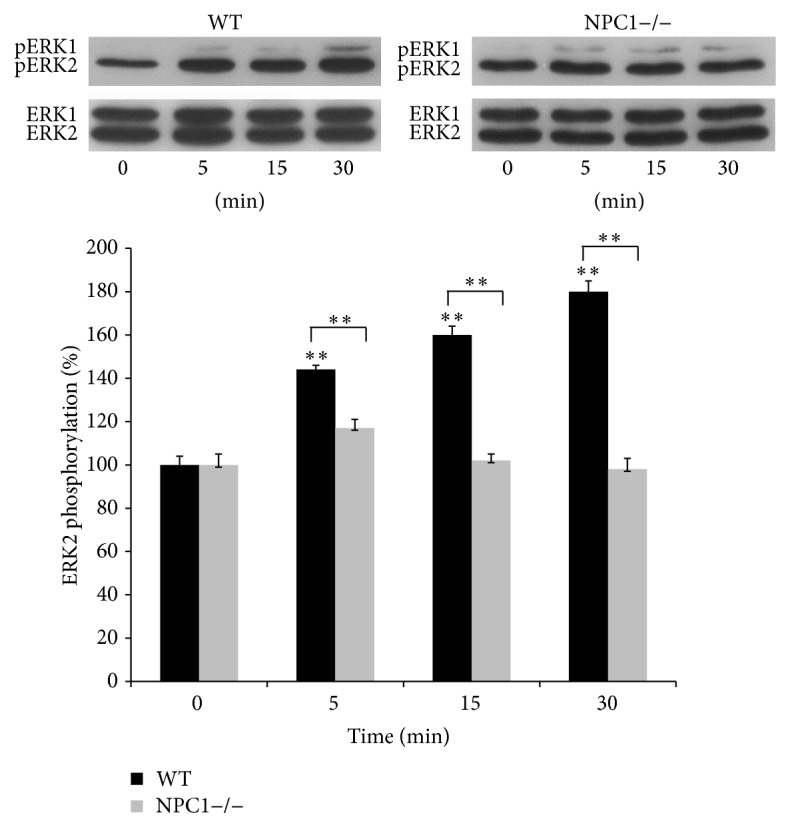
Time-course analysis of phosphorylation changes of ERK1/2 during LTP in WT and NPC1−/− mice. Representative Western Blots of phosphorylated and total form of ERKs are shown in the upper panels. Densitometric quantitation of the immunoreactive bands at different times after LTP induction is illustrated in the lower panel. Values represent the normalized percent changes in ERK2 protein phosphorylation for each time point after LTP. Bars in the plots represent means ± SEM (*n* = 6 for WT mice, ^*∗∗*^ *p* < 0.01 versus control values (*T*0); *n* = 6 for NPC1−/− mice, ^*∗∗*^ *p* < 0.01 versus WT mice at the corresponding time point).

**Figure 4:**
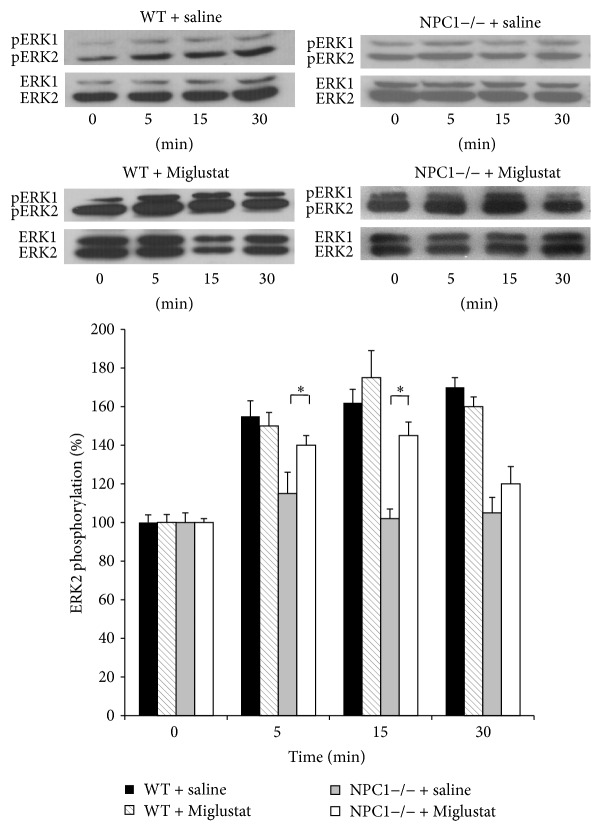
Effects induced by Miglustat treatment on the activation of ERK1/2 during LTP in WT and NPC1−/− treated mice. The immunoreactive levels of activated and total kinases at the indicate times are shown for a representative experiment in the upper panels; normalized values (means ± SEM) of activated kinases are illustrated in the lower panel (*n* = 5 for NPC1−/− mice treated with Miglustat; ^*∗*^ *p* < 0.05 versus untreated NPC1−/− mice at the corresponding time point).

ERK phosphorylation has been shown to occur following different LTP inducing paradigms in hippocampal slices [16]. Indeed, after LTP induction, ERKs are considerably phosphorylated and the extent of this phosphorylation depends on the type of the LTP inducing paradigm [17]. Here, we analyzed the phosphorylation state of ERKs at distinct time periods (5, 15, and 30 min) after delivering the high frequency tetanus. To this aim, a single slice for each time point (from a single mouse) was subjected to electrophysiology, rapidly dissected to isolate CA1 region before it was homogenised and protein lysates analyzed by Western Blot (Figure 3). Quantification of the immunoreactive levels of the activated ERK2 kinase, normalized by the amount of the total kinase, revealed that induction of LTP increased ERK2 phosphorylation in slices from WT mice by approx. 47%, 60%, and 80% at 5, 15, and 30 min after LTP, respectively (*n* = 6 from 6 different animals for each time point, *p* < 0.01). The same tetanic stimulation in the NPC1−/− mouse slices failed to induce a significant ERKs phosphorylation at each time point (*n* = 6 from 6 different animals, *p* < 0.01) (Figure 3). We then asked whether Miglustat was able to restore ERKs phosphorylation in stimulated NPC1−/− mouse slices. Therefore, we evaluated ERK2 phosphorylation levels in CA1 region of slices from NPC1−/− mice treated either with Miglustat or with saline at different time periods after LTP induction. We found that Miglustat reestablished ERK2 phosphorylation levels principally at 5 and 15 min after LTP (40% and 45% increment, resp., and 20% after 30 min; *n* = 5 from 5 different animals for each group *p* < 0.05, Figure 4). As control of gavage procedure and to verify the specificity of Miglustat action, we also evaluated ERKs activation in slices from WT mice treated either with saline or with Miglustat. We found that ERKs phosphorylation levels did not significantly change with respect to slices from untreated animals (*n* = 4 from 4 different animals for each group, Figure 4).

## 4. Discussion

In this study, we analyzed whether synaptic plasticity was altered in NPC1−/− mice, a well-established mouse model of the NPCD [12], and we proposed Miglustat as treatment to counteract the synaptic deficits. The main findings obtained from this study can be summarized as follows: (i) BST, which we previously found enhanced in NPC1−/− mouse slices, returned to normal values after Miglustat treatment; (ii) hippocampal PTP and LTP induction and maintenance were considerably reduced in slices from NPC1−/− mice whereas Miglustat* in vivo* administration was able to revert this impairment; (iii) application of a tetanic stimulation, which induced a rapid and strong increment in ERKs phosphorylation in WT hippocampal slices, failed to induce a significant ERKs phosphorylation in NPC1−/− mouse slices and Miglustat treatment was capable of restoring this activation; (iv) oral treatment of NPC1−/− mice with Miglustat was able to prevent the onset of symptoms* in vivo*.

Tetanic stimulation of Schaffer collaterals in CA3 area of the hippocampus of NPC1−/− mice induced a synaptic plasticity characterized by a partial inhibition of both PTP and LTP induction, whereas the maintenance phase was completely blocked. These results, obtained by recording the PS at the level of pyramidal layer, are partially in line with the observation of Zhou et al. [18], which demonstrated a defect in hippocampal LTP in NPC1 mutant mice with a reduction of field EPSP.

Posttetanic LTP in the hippocampal region has been widely studied and ascribed to the NMDA and AMPA receptor activity [19, 20]. The reduction of both PTP and LTP that we found in NPC1−/− slices could be caused by a malfunction of several different mechanisms correlated with these receptor activations. First, PTP in NPC mice could be affected by a decrease in neurotransmitter release due to a reduced elevation in presynaptic Ca2+ after tetanic conditioning. Indeed, several studies support the important role played by cholesterol and membrane rafts in the neurotransmitter release process. For example, it has been reported that exocytosis of synaptic vesicles, including Ca2-dependent glutamate release, is impaired following a decrease in cholesterol levels [21, 22]. Furthermore, in a previous paper, we provided evidence that agents interfering with plasma membrane cholesterol (methyl-beta-cyclodextrin (Cdex)) inhibit the NMDA-stimulated influx of calcium in hippocampal cells in culture [23]. Indeed, in another paper from our group, we demonstrated that Cdex strongly reduces synaptic transmission and blocks the expression of LTP [11].

The NPC1−/− mouse hippocampal neurons may present an impaired lipid domain organization that affects the NMDAR transduction pathway leading to an impairment of LTP induction. The hindering of the LTP maintenance phase could be due to a defective regulation of GluR1 AMPA receptor trafficking and a reduced exposure of these receptors on the cell surface as a consequence of the gangliosides and/or cholesterol accumulation. These results may be in agreement with Brachet et al. [10] who have recently demonstrated that NMDAR activation during LTP induction leads to a loss of redistribution of intracellular cholesterol in the neuron triggering AMPA receptor synaptic delivery and, in turn, synaptic potentiation.

Overall, these results indicate that a cholesterol dysmetabolism may be responsible for synaptic plasticity phenomena impairment, suggesting that the LTP impairment described in NPC1−/− mice could result from a defect in lipid transport system.

In WT mouse slices, we previously reported a rapid and strong increment in ERKs phosphorylation after delivery of LTP inducing HFS, which lasted for at least 30 min [14]. Application of a tetanic stimulation in NPC1−/− hippocampal slices was unsuccessful to induce a significant ERKs phosphorylation. Given the possibility of a reduced NMDA receptor activation dependent on cholesterol levels and thus a reduced Ca2+ influx in NPC1−/− slices, it is reasonable to hypothesize that the lack of ERKs phosphorylation may depend on a failure of the transduction mechanism mediated by NMDAR [24].

In the present study, we observed that Miglustat administration is able to revert both the hyperexcitability previously reported in NPC1−/− mouse hippocampal slices [15] and the impairment of PTP and LTP. Following the hypothesis that the mechanism by which Miglustat is able to revert the impairment of synaptic plasticity in NPC1−/− mice involves ERKs phosphorylation, we expected that* in vivo* Miglustat administration could restore ERKs phosphorylation in stimulated NPC1−/− mouse slices. Indeed, Miglustat treatment was capable of reestablishing ERKs activation in slices from NPC1−/− mice suggesting a direct effect of the drug on the signal transduction pathways of synaptic potentiation.

Since 2009 Miglustat has been designated Orphan Medicinal Product by EMA for the treatment of neurological symptoms in NPC patients. Indeed, several advantages have been noticed in clinics during Miglustat therapy and in particular amelioration of neurological impairment of NPC patients, mostly in adolescent or adult onset, before irreversible neurological damage occurs. However, the reason for this amelioration has yet to be determined.

Our observations regarding a protective effect of Miglustat on NPC1−/− mice (Video supplementary material) are in agreement with the data reported by other authors although in our study we used a low dose of the drug (0.2 mg/kg/die by gavage) [25, 26]. The choice of gavage administration was made to give the drug in the exact amount whereas, in other studies, Miglustat treatment was carried out by feeding animals mixing the drug in the pellet diet (50–1200 mg/kg/day).

*In vitro*, Miglustat acts as an inhibitor of glucosylceramide synthase, the enzyme that regulates the first step of ganglioside synthesis and for this reason has been used to ameliorate several dyslipidosis, including Gaucher and GM1 gangliosidosis. Depletion of glycosphingolipids by Miglustat treatment reduces pathological lipid storage, improves endosomal uptake, and normalizes lipid trafficking in peripheral blood B lymphocytes from NPC patients [27]. The fact that Miglustat, which has no direct effect on cholesterol metabolism, corrects the abnormal lipid trafficking seen in B lymphocytes from NPC patients may indicate that glycosphingolipid accumulation could be an important pathogenetic event in NPC disease.

## 5. Conclusion

It has been recently proposed that, beyond its effect on glycosphingolipids synthesis, Miglustat could play other roles such as interfering with histones acetylation [28], blocking the oxidative stress (observed in NPC cell patients), or acting as chaperon to give the correct folding of mutated NPC1 [29]. A better understanding of the mechanisms of action of Miglustat is critical to improve its therapeutic role.

## Supplementary Material

The video shows the different behaviours observed in NPC1−/− not treated and in NPC1−/− treated mice with Miglustat for 20 days. The treated animal does not show insurgence of neurological symptoms presenting a normal function motor system while the NPC1−/− not treated mouse does not move quickly to explore the cage, often it remains in the same position displaying ataxia and tremor.
